# OLDER ADULT ACCESS TO SELF-DIRECTED SERVICES THROUGH MEDICAID HOME AND COMMUNITY BASED SERVICE WAIVERS

**DOI:** 10.1093/geroni/igae098.3874

**Published:** 2024-12-31

**Authors:** Natalie Turner

**Affiliations:** University of Washington, Seattle, Washington, United States

## Abstract

Self-Direction (SD) is an underused service delivery model shown to improve health outcomes and increase care satisfaction for older adults. Rooted in choice, empowerment, and control over long-term services and supports (LTSS), SD epitomizes person-centeredness. And, because of its ability to provide LTSS in a culturally responsive manner, has the potential to enhance racial equity in LTSS provision. Since each state determines what services can be self-directed and how many beneficiaries can self-direct, state policy impacts access to and use of SD. This mixed-method study identified state differences in access to SD among 1915(c) waivers that serve older adults using Framework Analysis approach and descriptive statistics. Findings indicate potential access barriers to SD provided through 1915(c) waivers. Of the 60 waivers serving older adults in the U.S., 35 allowed SD (58%), representing 26 states and DC. While states described the benefits of SD within waiver applications and highlighted its alignment with person-centeredness, most set very low goals for the number of beneficiaries expected to use SD. Seventy-five percent of waivers set goals for participation in SD at less than 25% of beneficiaries. Furthermore, states acknowledged SD’s flexibility and ability to tailor services to participant needs, while limiting the types of services individuals could self-direct. In light of the established benefits of SD, these findings raise questions about potential underutilization of SD among older adults. Further research is needed to better understand apparent discrepancies between state descriptions of SD and perceived and actual access to these services.

